# Telehealth use for sexual and reproductive health promotion and care during the early phase of COVID-19 pandemic: A descriptive-interpretive qualitative study of healthcare providers’ perspectives and experiences in Western—Central New York State

**DOI:** 10.1371/journal.pgph.0003259

**Published:** 2024-12-23

**Authors:** Sadandaula Rose Muheriwa-Matemba, Danielle C. Alcena-Stiner, Alexander Glazier, Natalie M. LeBlanc

**Affiliations:** 1 Human Development Nursing Science, College of Nursing, University of Illinois Chicago, Chicago, Illinois, United States of America; 2 School of Nursing, University of Rochester, Rochester, New York, United States of America; 3 Medical Center, University of Rochester, Rochester, New York, United States of America; 4 Center for Interdisciplinary Research on AIDS, School of Public Health, Yale University, New Haven, Connecticut, United States of America; The Chinese University of Hong Kong, HONG KONG

## Abstract

Telehealth emerged as a key option for the provision of sexual and reproductive health (SRH) care and promotion during COVID-19 pandemic restrictions. However, there is limited research on the perspectives and experiences of healthcare providers (HCPs) in the Western-Central region of New York State. This qualitative interpretive study explored the perspectives and experiences of the HCPs’ with telehealth for SRH promotion and care including counselling, testing and treatment for HIV infection and other sexually transmitted infections (STIs), in Western New York State. Ten HCPs participated in semi-structured in-depth interviews conducted between October 2019 and February 2021. Participants were predominately White, female, with 1–30 years of clinical experience. The narratives revealed three major themes: 1) HCPs’ perspectives of telehealth use, 2) HCPs’ experiences with telehealth use for SRH promotion and care, and 3) determinants of telehealth implementation. Though all providers reported an increase in the use of telehealth, experiences in the delivery of telehealth varied especially for SRH services. Some providers reported having more time to consult with patients because patients could just call and schedule a telehealth visit and because of a decrease in patient load which freed up time to engage with patients. Others reported technological limitations among some patients which impacted care. Strengthening telehealth-based sexual health promotion will serve to address efforts toward ending the HIV epidemic, reducing other STIs, and ensuring consistent access to contraception. To effectively implement telehealth findings, suggest a need to ensure adequate technological resources for patients, and a need to increase HCPs’ comfort to engage patients in sexual health conversations via telehealth.

## Background

Public health responses to the COVID-19 pandemic were unprecedented in modern times regarding its impact on healthcare delivery [1,2]. Quarantines, institution-wide closures, and reallocation of resources towards emergency services compromised routine sexual and reproductive health (SRH) services [3–5]. Global disruptions in HIV prevention and care were associated with 650,000 HIV-related deaths [510,000–860,000] and 1.5 million [1.1–2.0 million] new HIV infections in 2021 [6]. Moreover, a 2022 scoping review by VanBenschoten et al [7] revealed that 86% of SRH clinicians and stakeholders in 29 countries reported that patients had much less access to SRH services due to the pandemic. In the United States (US) the report identified an 80% decrease in HIV preexposure prophylaxis (PrEP) prescriptions. Other US studies reported a stark increase (33%) in maternal deaths after March 2020, albeit some resulted from direct viral infections, while others have been attributed to the disrupted healthcare systems [8]. More than half of clinics canceled or postponed contraceptive visits, and 21% of SRH clinics in the South and Midwest parts of the country closed per COVID-19 pandemic mandates [7]. In addition, electronic survey results from a California-based sexually transmitted diseases (STDs) control branch found sharp declines in reportable bacterial cases of chlamydia (31%), late syphilis (19%), primary/secondary syphilis (15%), early non primary non-secondary syphilis (14%), and gonorrhea (13%) between 2019–2020 [9].

As the COVID-19 response intensified, telehealth services emerged as an option for accessing health care services, particularly those services considered non-essential to maintain healthcare delivery in the US and globally [10,11]. Telehealth, defined as the use of telecommunications and information technology including computers and mobile devices to provide access to health assessment, diagnosis, intervention, consultation, supervision, and information virtually [12–14], enabled patients to access support and clinical care from any location. E-health refers to health services and information delivered or enhanced through the internet and related technologies [14,15]. Telehealth in combination with e-health helped healthcare providers (HCPs) to track and manage the health conditions of patients remotely to mitigate COVID-19 transmission [16,17]. An internet-based study among cisgender women in the US found that telehealth helped to fill in some new SRH service gaps [11]. Due to the pandemic, 24% of women using contraception switched to a telehealth appointment to have prescriptions refilled. Telehealth has been reported to be patient-centered, conducive to self-quarantine, and protecting patients, clinicians, and the community from exposure to infection [10]. More frequent check-ins through telehealth and remote patient monitoring also helped HCPs identify some complications faster, leading to fewer hospitalizations and emergency room visits [16,17].

Telehealth service delivery during COVID-19 pandemic restrictions has been offered in different ways with live video teleconferencing being the most popular modality [18,19]. Other modalities included store-and-forward technology which is the transmission of a patient’s medical information to a HCP at a distant site [20], remote patient monitoring such as telehealth coverage of intensive care units, and mobile health applications including text, and email [16,21]. In the US, most facilities have been reported to use a combination of telephone and video for telehealth visits [18,19]. Many HCPs also used Health Insurance Portability and Accountability Act (HIPAA) compliant applications to conduct virtual telehealth visits which assured patient privacy [19].

In the US, insurable services and processes, health policy, and practice may vary regionally. Therefore, HCPs’ perspectives across state and county levels are needed to address the heterogenous subtleties in telehealth utilization for SRH promotion. Moreover, the experiences of HCPs in Western-Central New York State in their provision of SRH promotion and care during the COVID-19 pandemic has not been well documented. When New York State emerged as one of the epicenters for COVID-19 in 2020, SRH services were significantly reduced with non-essential services closing as of March 22, 2020, while hospitals around the state were encouraged to prioritize their resources to address the pandemic [4]. This impaired facility-based services and in-person health visits. Nevertheless, HCPs were encouraged to promote SRH, conduct a complete sexual health history, and assess susceptibility to sexually transmitted infections (STIs). An advisory from the New York State Department of Health also offered strategies for HCPs and community-based organizations to support SRH and prevent STIs during COVID-19 restrictions [22]. Understanding the experiences of HCPs’ delivery of SRH services in tandem with the uptake and use of telehealth during COVID-19 pandemic is warranted. Such insights can inform the suitability of telehealth for SRH promotion and care, and implementation needs for sustainability of this modality in Western-Central New York.

## Methods

### Study design

This study used a qualitative descriptive-interpretive design [23] to understand the perspectives and experiences of HCPs with the use of telehealth for SRH promotion and care during the COVID-19 pandemic. A qualitative descriptive interpretive approach allowed the investigators to address complex experiential SRH promotion questions during COVID-19 pandemic, while understanding practical outcomes and underlying experiences. The researchers wanted to go beyond reporting the findings and interpret what the findings meant in the study context [24]. This approach allowed the advancement of knowledge and experiences surrounding SRH promotion, care, and support during the COVID-19 pandemic without sacrificing methodological integrity that long-established qualitative approaches provide [24].

### Sample and sampling

Semi-structured in-depth interviews were conducted with 10 HCPs who had previously been interviewed about sexual health promotion and couple-centered approaches. This sample size was determined by saturation. These providers were a subset of this group and were practicing in women and adolescent health clinics, HIV/STI testing and care settings and family medicine clinics. Thus, the participants had previously interacted with the researcher in the first phase of the study. These HCPs were recruited from October 2019 to February 2021 using maximum variation purposive sampling [25]. The procedures and the analyses of this study were approved by the University of Rochester Institutional Review Board.

### Recruitment

Active and passive recruitment strategies were used, which included circulating study flyers via emails to university research listservs, posting flyers in the health care facilities and on social media, and meeting providers at department or facility grand rounds to discuss the research. Subjects were asked to either contact the study team directly or complete a screener form in REDCap to assess their eligibility. Eligibility criteria included being over 18 years old, speaking and understanding English, and being a licensed clinician practicing in New York State with one or more years of experience in SRH. All eligible participants were provided with an information letter that contained study details. Upon reading the information letter in REDCap, providers indicated their desire to participate in the study, and they all provided written consent. Interested participants were asked to provide their socio-demographic and contact information. Upon receipt of the notification of completion of the REDCap survey, the study staff followed up with a phone call and scheduled the interview. None of the participants sampled in this subset of the sample refused or dropped out during the interview. The interviews were conducted by the last author NML, a female PhD prepared Assistant Professor and an interdisciplinary nurse researcher, with over 20-years of career in public health, nursing, and health research. NML uses multi-method qualitative/quantitative approaches to inquiry, and investigates the ecological, cultural, and systemic factors (social determinants of health) that influence health and wellness outcomes. As a public health specialist, NML is able to critically assess health issues from both public health and clinical perspectives globally and domestically. As a nurse researcher, NML seeks to address and investigate determinants of health disparity, assets within these factors that can be leveraged toward achieving health equity to inform intervention implementation. At the beginning of each interview, the interviewer re-introduced herself and the purpose of the study and verified their interest to participate in the study. All interviews were conducted via Zoom, in a private room with only the participant present.

### Data collection

The individual in-depth interviews were guided by a semi-structured interview guide which was piloted on one participant and repeated on another one participant after making the corrections to the interview guide. Each semi-structured in-depth interview concentrated on the experiences of the participants with SRH promotion during the COVID-19 pandemic. This report is focused on the use of telehealth. Participants were asked the following main questions (with follow-up prompts that are not listed):

1. Describe the impact of the COVID-19 pandemic and quarantine on your personal practice in sexual health promotion with patients.
2. What changes have you made to your personal practice in sexual and reproductive health, HIV/STI prevention, and/or care promotion during the COVID-19 pandemic?
3. Describe your experiences with using telehealth during this time, including what worked well for you as a provider and for your patients?

The interviews lasted 30–45 minutes and each participant was paid 30 US Dollars for their time. All interviews were recorded with HIPAA compliant University of Rochester Medical Center Zoom and iPad. Field notes were also taken to capture the necessary contextual information. Data were transcribed verbatim by an outsourced transcription company.

### Data analysis

The thematic analysis involved an inductive iterative approach to generating codes, themes, and subthemes [26]. All themes and subthemes were generated from the data. Upon transcription, the first, second, and senior authors read each transcript at least 3 times line-by-line to achieve immersion into the data and obtain a sense of a whole while identifying the underlying concepts and clusters of concepts [26,27]. These authors read each transcript, highlighting text that was salient and appeared to describe perspectives and experiences with SRH promotion and care; and using telehealth during the COVID-19 pandemic. Memos noted initial impressions, as well as keywords or phrases that captured the perspectives and experiences of health providers. Memoing allowed a reflection on findings to deepen our analysis, generate new ideas and interpret and communicate our findings. Memoing also assisted in making conceptual connections from raw data to those abstractions that explained the use of telehealth for sexual health promotion during the COVID-19 pandemic [28]. Via an iterative process to create a coding schema that reflected critical thoughts and codes that came directly from the interpretation of the interviews. This process was facilitated by MAXQDA 2020 software [26,27,29].

After coding all transcripts, the investigators conducted peer debriefings where they examined and developed each code and refined the descriptive statements for each code. This process led to combining some codes to form main themes and splitting others into sub-themes based on how different codes were related and linked to each other. These emergent themes helped to organize and group codes into meaningful clusters [30,31], while keeping them broad enough to sort a large number of codes [32]. Depending on the relationships between sub-themes the investigators combined and organized the more substantial number of subthemes into a smaller number of themes. Next, the definitions for each theme and sub-themes including the text segments that corresponded to each theme were documented in the coding book [33]. Themes and subthemes were then organized [29] to illuminate health care providers’ perspectives and experiences with SRH promotion and telehealth use during the COVID-19 pandemic. Peer debriefing was conducted to ensure that there was agreement or reconciliation with the findings, coding, and thematic scheme. Later findings were presented to the participants for them to verify the results.

## Results

Table 1 displays the sociodemographic characteristics of the participants. The participants were mainly White, female, and had varying years of experience (1–30 years).

**Table 1:** Socio demographic characteristics of the participants, (*N* = 10).

Sociodemographic Characteristic	*Mean*	*Range*	*n (%)*
Age	51	28–67	
Sex			
Male			4(40)
Female			6(60)
Ethno-race			
Non-Latino Black/ African American			1(10)
Non-Latino White			7(70)
Latino			1(10)
Other			1(10)
Religion			
None			3(30)
Christian			7(70)
Type of health profession			
Medical Doctor			5(50)
Advance Registered Nurse Practitioner			2(20)
Nurse Midwife			1(10)
Physician Assistant			2(20)
No. of years in the health profession	21.3	2–43	-
No. of years the HCP has been licensed in the profession	23.1	2–43	-
Type of facility the HCPs worked			
Public Clinic			2(20)
Federally Qualified Health Center			3(30)
Private & Academic health facility			3(30)
Hospital based clinic			1(10)
Outpatient clinic and juvenile detention center			1(10)

**Fig 1:**
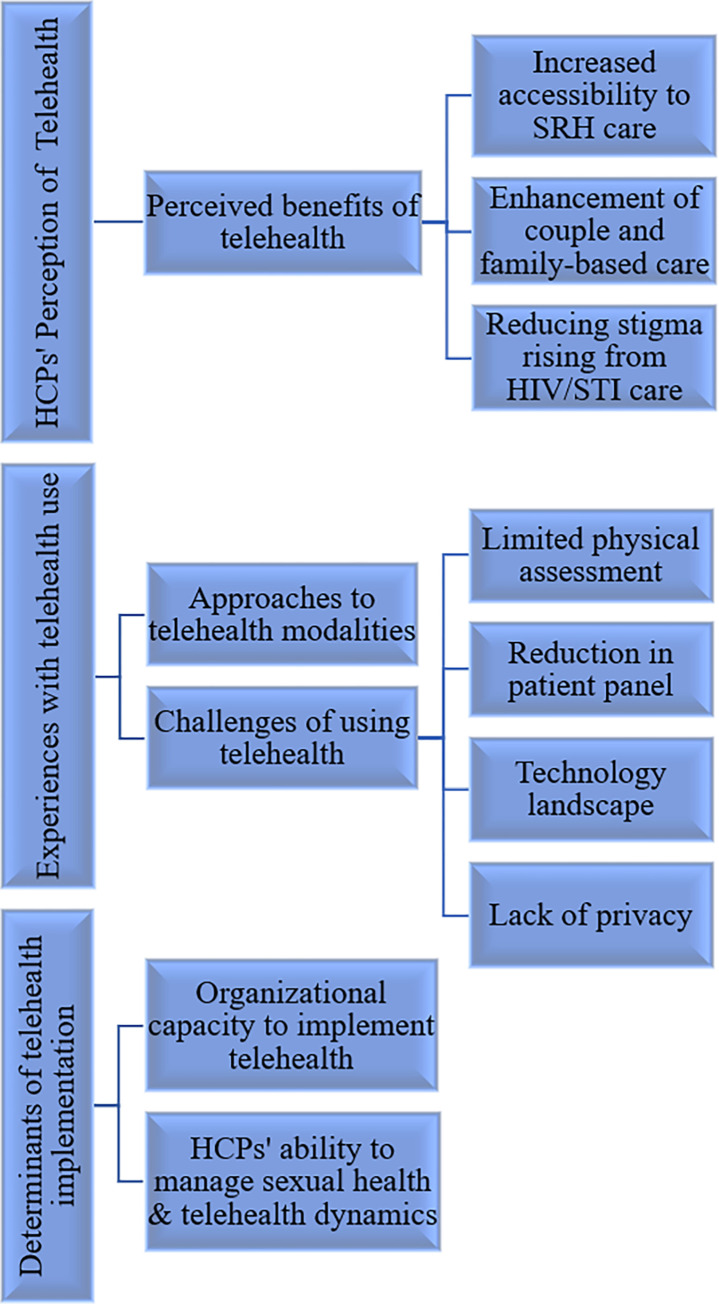
Themes and sub-themes emerging from the healthcare providers’ narratives.

Narratives from participants revealed three key themes related to the use of telehealth for SRH promotion and care: 1) healthcare providers’ perspectives of telehealth use, 2) healthcare providers’ experiences with telehealth use for SRH promotion and care, and 3) determinants of telehealth implementation. Fig 1, illustrates the themes and sub-themes emerging from the HCPs narratives.

### Theme 1: HCPs’ perceptions of telehealth

Narratives revealed HCPs’ perception of telehealth use for SRH promotion and care. Participants reported their perceived benefits of using telehealth despite some limitations during inception. Their perspectives were in part influenced by telehealth as a modality, their experience with telehealth uptake in response to the COVID-19 restrictions, and due to organizational considerations for the adoption of telehealth service. Perceptions were also influenced by patient capabilities to engage with the technology. One participant said:

“*The more you make it [telehealth] available and decrease the sort of limitations to access, uh, it seems to be more successful and widely used* (Nurse-Midwife, > 25 years of being professionally licensed). Another participant said: “*it [telehealth] may improve access for people as long as you can work out some of the other logistics”* (Physician Assistant, <10 years of being professionally licensed).

#### 1.1 Perceived benefits

The narratives revealed three main benefits of using telehealth during the COVID-19 pandemic: increased accessibility to SRH care, enhancement of couple and family-based care and reducing stigma and discrimination.

*1*.*1*.*1 Increased accessibility to SRH care*. Providers reported that telehealth use increased the accessibility of care for HIV infections and other STIs during the COVID-19 pandemic. One participant said:

*“It [use of telehealth] has become a little bit easier to talk to people together with telemedicine. Somebody can kind of just casually join a visit, even if they’re not on the schedule, which is not something that’s possible, you know, in an office setting. That’s actually been kind of nice”* (Physician Assistant <5 years of being professionally licensed).

*1*.*1*.*2 Enhancing couple and family-based care*. Regarding the benefit of promoting the provision of couple and family-based care, the HCPs reported that telehealth expanded SRH services to populations that were difficult to reach with in-person services. According to the HCPs, telehealth enabled individuals to share testing kits with their sexual partners, there by expanding STI screening within sexual networks and potentially improving public health outcomes. The HCPs reported the practice of using telehealth for sexual health services, such as distributing at-home testing kits, gained momentum, and become more widespread during the COVID-19 pandemic. One participant said:

*“At least in New York state, telehealth has been shown to not only increase testing to populations that weren’t previously reached by in-person, but also, it seems to increase testing in sexual networks because you can have a kit and maybe you can have a second one and give it to your partner. I feel like all those things have sort of taken flight”* (Midwife, >30 years of being professionally licensed).

*1*.*1*.*3 Reducing stigma rising from HIV/STI care*. Participants also perceived that telehealth use for patients helped to ease engagement with the healthcare system, and potentially addressed stigma arising from HIV/STI health care visits. One participant said:

*“Because you [patients] could request a kit and not have to come in contact with a health care personnel. This helped to overcome part of the stigma associated with seeking care for HIV and STIs because you’re able to do something online. The process is anonymous. Even if it may not be, but it sort of takes away that step of having to go somewhere in person”* (Medical Doctor, >25 years of being professionally licensed).

### Theme 2: Experiences with telehealth use

For most providers, the emergent use of telehealth and e-health was in the context of the health facilitys’ response to the COVID-19 pandemic. Providers’ perspectives and experiences with telehealth, especially in the context of sexual health (including HIV/STI testing and care) varied and was in part influenced by how health facilities responded to organizational accommodations to COVID-19 restrictions. Statewide COVID-19 restrictions required healthcare facilities to re-assess their provision of what was considered non-essential health services. This included routine SRH promotion and care including HIV and STI prevention, diagnosis, and treatment services. Re-assessment entailed either healthcare services being exclusively redirected to address the health needs resulting from COVID-19 pandemic; provision of essential health services in addition to COVID-19 specific care; or exclusive modification of sexual health services to remote telehealth services with occasional triaged in-person follow-up visits. This modification resulted in some instances, halting and slowing the provision of sexual health services. In other instances, services remained intact with the addition of telehealth.

*“We pivoted to telemedicine visits, mail-in pharmacy, doing telehealth visits and STI testing by sending testing materials, swabs, instructions, and lab materials and requisitions to people at home so that they could collect specimens at home, drop them off in the lab. In other words, trying to make this as seamless as possible and continue what we consider essential services. We also opened a COVID-19 testing area that is still in operation”* (Medical Doctor, primary care, > 40 years of being professionally licensed).

The HCPs also reported that patients with SRH problems and needs were deprioritized when scheduling appointments. This happened mainly in the early days when HCPs had not much information about COVID-19 pandemic. Priority was given to COVID-19 prevention and care, and the implementation of telehealth served as a crucial solution for patients with SRH problems and needs to access care. Some providers adopted telehealth after gaining a better understanding of COVID-19 transmission from both epidemiological and clinical perspectives.

*“We do pregnancy and then gynecological care. We were limited as to how many people we could bring in. Patients that were having gynecological problems, STDs or needed contraception, they kind of took a back seat to what was available as far as appointments. So, we were able to offer them telehealth that helped them access care”* (Advance Registered Nurse Practioner, women’s health, > 25 years of being professionally licensed).

Overall, the HCPs reported positive experiences with telehealth use. The participants also reported of how they found telehealth to be a feasible and acceptable modality of providing care during the pandemic. Positive experiences were related to provider ease and comfort not only with modality, but also with the capability to have their patients’ sexual health needs met. There were cases where providers reported that telehealth facilitated the adoption of certain services like at- home HIV/STI tests, and patient capability to self-collect specimens. This modality helped with continuity of care in the provision of sexual health services.

*“I think the transition has gone reasonably well and, and people like the option of having some telehealth visits. Tele PrEP is probably a pretty good thing. And I think it may improve access for people as long as you can work out some of the other logistics”* (Physician Assistant, <10 years of being professionally licensed).

Another participant also said:

*“The people I’ve talked to seem to like it [telehealth]. They like doing their own testing that includes HIV testing and genital swabs. And people are doing their own rectal swabs and throat swabs, and they’ve learned to do it and are comfortable with it. I was, um, pleasantly surprised at the comfort level and they accept it”* (Medical Doctor, >25 years of being professionally licensed).

#### 2.1 Approaches to telehealth modalities

Providers reported on approaches to telehealth in the context of the agency’s capacity in tandem with the health facility’s focus on HIV/STIs prior to the onset of the COVID-19 restrictions. Providers reported use of different approaches to telehealth use which included telehealth by appointment, walk-in video visits and other web-based modalities such as PrEP2Me.

*“It’s really like making it readily available where you can meet people where they are. In some places you can only do it by appointment, so that’s very restrictive. Here, you can just call in, get scheduled and go through a visit at any time during the time the clinic is open. There are other places where it starts as kind of a web-based thing. So, you just submit your information and then they reach out to you directly”* (Nurse-Midwife, > 25 years of being professionally licensed).

Providers’ reports also indicated that video telehealth was the most preferred modality for telehealth visit as it allowed them to see and physically assess their patients. The narratives revealed the importance of observing the non-verbal cues via video during telehealth because it helps to confirm diagnosis and care. The HCPs reported that when not on camera, it is easy for patients to just say they are doing well yet, they are not. One participant said:

*“I saw somebody recently. We asked how the person was doing, and they said fine, but on the video, they’re crying. So, you know, on the phone, I wouldn’t see that because you couldn’t hear it in the voice necessarily”* (Physician Assistant, <5 years of being professionally licensed).

#### 2.2 Challenges of using telehealth

Providers reported challenges they faced with using telehealth. These included: loss to follow up, lack of opportunity to examine the patient, internet interruption, scam calls that made the patients not respond to real phone calls for the hospital visit, lack of privacy, and lack of time management and seriousness by the clients.

*2*.*2*.*1 Reduction in patient panel*. While some providers reported an increase in patients who normally would not engage, other providers reported that in-person restrictions resulted in a reduction in patient volume and patients’ loss to follow-up.

*“We did lose about 10% of our 1000 patients though who drifted away and have been a little harder to reconnect. But overall, I think it certainly was preferable to not doing anything. And we made an effort to stay connected with, uh, with people”* (Medical Doctor, > 25 years of being professionally licensed).

As some providers report and increase in HIV tested via telehealth other providers reported a reduction in patients as a result in restrictions on their practice. One participant said: *“there was probably decreased access*, *not because we weren’t open*, *but because patients weren’t coming in to get tested”* (Medical Doctor, 10 years of being professionally licensed).

*2*.*2*.*2 Limited physical assessment*. Providers reported that while telehealth engaged patients, it lacked the opportunity for physical assessments, which they considered an essential aspect of sexual health promotion visits. The inability to conduct a physical examination was a significant limitation of telehealth. One participant said:

*“Yeah, sometimes telemedicine tends to be, um, more challenging because you can’t examine a person and some people do need an exam, so that, you know, you do have to defer that to a different setting, but, um, it really is well suited to sort of preventive care or well care”* (Nurse Midwife, >25 years of being professionally licensed).

Another participant added:

*“There are just some situations where you can’t do it by video. When you need to touch the patient and you can’t. Or listen to the patient’s lungs, or if they had a rash. We would attempt to set them up for a video appointment because you can describe a rash as best you can. But I mean, that visual is what makes the diagnosis”* (Medical Doctor, 10 years of being professionally licensed).

*2*.*2*.*3 Technology landscape*. Internet interruption was another challenge that the participants experienced particularly with those clients of low socioeconomic status.

*“Sometimes it just doesn’t work. They just don’t have the Wi-Fi, and it’s not very good. And so we have to end up turning off the video to hear what the person’s saying. Lack of internet or lack of any type of video resource pose the challenge”* (Physician Assistant < 10 years of being professionally licensed).

The participants also reported on challenges posed due to lack of familiarity with the technology for both providers and patients. Both providers and patients faced difficulties in adapting to telehealth services, highlighting the challenge of transitioning from in-person visits to virtual appointments.

*“It was like a big adjustment to me, more so a challenge, I guess, like for the patients, just understanding that you know, this is a telephone appointment, or this is a video appointment and getting them, the proper resources that they needed to be prepared for a telephone or video appointment”* (Medical Doctor < 10 years of being professionally licensed).

Providers also reported that patients at times would not answer hospital calls thinking that they were calls from scammers. In response, some health facilities introduced phone applications that helped to overcome this challenge.

*“We use, Doximity. It’s targeted towards clinicians. The phone number shows up, which is really important on their phone. So now they’re answering our phones. They basically click through a series of allows. It’s HIPAA compliant”* (Physician Assistant, <10 years of being professionally licensed).

*2*.*2*.*4 Lack of privacy*. Providers reported concern with privacy during telehealth consultations. Providers reported that patients were not always in a private location during the time of the telehealth visit consultation.

*“So, you do get into these dicey issues, and even though when people say that they’re in a safe space, I sometimes wonder and, uh, you know, confidential space, uh, you know, when you’re out in public or what have you, you know, how confidential is it?”* (Physician Assistant, <5 years of being professionally licensed).

Although some providers perceived couple or family-based telehealth visits favorable, others had some concerns. Some providers expressed uncertainty of whether they addressed patients’ needs if they sensed someone else like a family member was present in the home during the telehealth visit.

*“Here we separate partners for most of the visit, to ensure people are comfortable and sharing confidential information, because here we have a lot of privacy rules. But when you’re doing telehealth you can’t, um, you’re not always sure that you’re getting the full story, um, because there’s no sort of confidentiality”* (Nurse Midwife, > 25 years of being professionally licensed).

### Theme 3: Determinants of telehealth implementation

Implementation of telehealth was determined by providers’ experiences of the organizational capacity to facilitate telehealth, the HCPs’ ability to manage sexual health and technology dynamics.

#### 3.1 Organizational capacity to implement telehealth

The HCPs reported on prior knowledge and experiences with telehealth as one of the determinants for telehealth implementation. The HCPs, particularly those with experience of using telehealth prior to the pandemic reported positive experiences of using telehealth and the ease of rolling out telehealth for sexual health promotion. One participant said: *“Well*, *the telemedicine works well*. *We have been experimenting with that a little bit*, *prior to that [COVID-19 pandemic]*. *And so we had a little bit of a foundation and then just turned it”* (Medical Doctor > 25 years of being professionally licensed).

Organizational capacity to implement telehealth rested on several factors as reported by providers. One factor was the ability to reimburse for the provision of healthcare services via a telehealth visit. Providers reported that the insurance companies would only reimburse for the video-based telehealth visits and not phone-based visits. Providers reported that the insurance companies’ reluctance to reimburse telehealth visits by phone was a major barrier that made care for some patients inaccessible. Some patients did not have smartphones or WIFI to be able to have a video telehealth visit. One participant said:

*“I don’t think it’s fair to assume that every patient has an iPhone or a smartphone. There are patients that need only phone visits, and cannot afford video calls. And to make primary care sustainable, you really have to be able to care for patients in multiple ways. So, to assume that all patients have access to technology is inequitable”* (Advanced Registered Nurse Practioner >10 years of being professionally licensed).

Another participant added:

“*Obviously, we want video, it’s better, you get better quality assessment, but if the person can’t do it, what are you going to do? And why should the health center take the hit financially? Because we’re meeting the patient with the technology they have. It’s not gonna be sustainable to do phone visits based on what many of the payers are reimbursing”* (Physician Assistant <10 years of being professionally licensed).

#### 3.2 The HCPs’ ability to manage sexual health and technology dynamics

Providers reported a number of personal and organizational strategies that facilitated implementation of telehealth for SRH promotion and care. HCPs reported of their adaptation to pandemic restrictions and their understanding that organizational financial loss may be inevitable. Other providers reported that instead of physicians or Advanced Nurse Practitioners providing all services, the organization leveraged an interdisciplinary clinical team and optimized sexual health service provision by nurses and social workers.

*“So, ultimately primary care will not survive if it’s not paid for adequately by insurers. We have always worked to use things like nurse care management as part of PrEP and part of STI care. Moving that forward a little bit and really using, like, nurse phone calls with patients instead*” (Advance Registered Nurse Practioner >10 years of being professionally licensed).

Another participant added:

*“Having our social worker who follows up patients has facilitated the telehealth. She typically does follow-ups to make sure that the patient’s partner has been treated. Um, but she also follows up in the meantime too, especially if the patients tend to miss their appointment”* (Physician Assistant, <10 years of being professionally licensed).

## Discussion

This study explored the HCPs’ experiences with SRH promotion and care during the COVID- 19 pandemic. Findings revealed three major themes regarding telehealth use for SRH promotion and care: 1) healthcare providers’ perceptions of telehealth, 2) experiences with telehealth use, and 3) determinants of telehealth implementation. Findings highlighted the significant impact of telehealth service delivery across the Western and Central regions of New York state. Throughout the pandemic, SRH promotion and care was not regarded as essential, consistent with the previous studies [3,34,35]. Despite the critical importance of SRH services, the pandemic underscored a troubling perception of SRH as non-essential, highlighting systemic challenges in prioritizing SRH services during public health crises. However, while the SRH services were considered non-essential, the rapid shift to telehealth services effectively bridged gaps in care access across the Western and Central regions of New York state. HCPs in this study transitioned from in-person visits to new appointment modalities including the use of mail-order pharmacies, and home STI /HIV testing. In this study, this significant shift in care provision revealed that telehealth not only maintained continuity of SRH promotion and care during the COVID-19 pandemic and expanded service outreach to underserved populations. This finding highlights the need for healthcare systems to adapt to this new model of service delivery model as it can lead to a more flexible and patient-centered care, especially in underserved regions.

In this study, participants expressed a generally positive outlook of telehealth. Increased accessibility to SRH care emerged as a significant benefit, allowing patients, particularly those in underserved areas, to receive SRH services without the barriers posed by travel or scheduling during the time of COVI-19 restrictions. Additionally, providers noted that telehealth enhanced couple and family-based care, enabling a more inclusive approach to treatment and counseling. This flexibility not only supported individual patients but also fostered family involvement in care decisions. Importantly, HCPs reported that telehealth helped reduce the stigma associated with HIV/STI care. By providing a more private and convenient option for accessing services, telehealth encouraged individuals who might otherwise avoid care due to stigma to seek necessary support. Also, home- based testing may have helped increase STI screening within sexual networks and help linked people to care including PrEP care [36,37]. These findings might explain why as testing for HIV and other STIs reduced significantly globally due to the COVID-19 pandemic [38], in Western New York State, the testing services increased and new STI diagnoses increased by 77% [22]. These findings suggest the need for continued integration of telehealth in SRH promotion and care. The positive outcomes associated with telehealth during the pandemic highlight the necessity for healthcare systems to continue integrating these services into standard practice. This integration should include the development of supportive policies and funding that prioritize telehealth as a permanent option for SRH promotion and care.

The experiences of the HCPs also revealed some barriers to telehealth use among HCP in SRH. General barriers were a lack of provider comfort to manage sexual health dynamic and socioeconomic health disparities. This finding brought to light existing socioeconomic disparities that impact access to SRH services, emphasizing the need for targeted interventions to ensure equitable healthcare delivery. In addition, the HCPs also encountered difficulties, such as technological challenges for patients and providers and varying levels of provider comfort in discussing sensitive topics via telehealth. These impediments have also been reported in previous studies [18,19,39] and are indicative of telehealth being a relatively new mode of care delivery and can be foci for training current and future providers. Specifically, these findings revealed a need for training and resources to help healthcare providers feel more comfortable managing sexual health issues through telehealth. Empowering providers with the necessary skills and knowledge can enhance the quality of care delivered and improve patient outcomes.

Providers also reported several challenges with the implementation of telehealth, including loss to follow up, limited physical assessment, internet interruptions, and lack of privacy. Loss to follow up could be mitigated by ensuring clients have access to reliable and affordable internet, devices with video and audio capabilities, noise canceling such headphones and private spaces for consultations. Successful telehealth implementation also requires adjusting behavioral expectations for engagement such as making direct eye contact by looking at the camera, rather than at the screen displaying the person [40]. Clients who look away may be perceived as disengaged, which could affect HCPs’ interpretations of their mood or affect; however, this may simply result from technical issues like camera and screen positioning. Additionally, HCPs have noted challenges in assessing clients’ skin conditions during virtual consultations. To address this challenge, resources and guidance on clothing choices and lighting conditions may be needed, as these factors can affect the appearance of conditions during virtual physical assessments. Providing clients with telehealth resources, including the necessary equipment, could help optimize the effectiveness of these visits [40].

Promoting telehealth etiquette is essential to minimizing unintended interruptions and distractions, such as the choice of background images, filters, attire, or conducting visits from public locations. While clients may voluntarily disclose aspects of their environment or mention that they are speaking candidly in the presence of others (whether on or off-camera), HCPs may feel uncomfortable or distracted during the encounter. This underscores the importance of incorporating practice guidelines that balance the input of both clients and HCPs, ensuring that telehealth etiquette expectations are mutually understood and respected. The perspectives of HCPs in this study align with other reports, highlighting the need for “screen-side etiquette” during telehealth assessments and care. Such guidelines can help optimize the virtual setting (e.g., visual and audio quality) and reduce distractions from either the healthcare setting or the client’s environment [41].

The collective perceptions of HCPs in the current study highlight the lack of infrastructure preparedness and training which have hindered the broad adoption of telehealth modalities. Prior to the pandemic, research had already revealed the underutilization of telehealth training in both primary and specialty care settings for HCPs nurse practitioners and students in Nurse Practitioner programs. This gap emphasized the need to enhance providers’ comfort, knowledge, and skills to effectively embrace telehealth as a central component of healthcare delivery [42].

The findings also highlighted key determinants of successful telehealth implementation. A recurring theme was the organizational capacity to effectively integrate telehealth, with providers emphasizing the importance of institutional support, resources, and training. Furthermore, the ability to navigate the complexities of sexual health and telehealth dynamics was critical. This included not only technical proficiency but also comfort in discussing sensitive topics in a virtual environment. Enhanced training and ongoing support can empower providers to manage these dynamics more effectively, leading to better patient interactions and outcomes.

Several factors facilitated the implementation of telehealth for sexual health promotion. Organizational-level factors included the health facility’s capacity and preparedness in tandem with the existing plans to introduce telehealth before the COVID-19 pandemic, played a significant role. Facilities with pre-existing plans were able to expedite their implementation and strategically support clients in adapting to the new approach to remote sexual health promotion and care delivery. In contrast, the rapid increase in telehealth use during the pandemic, without established governmental and organizational guidance, was seen more as a pandemic response than a deliberate shift in practice. This suggests that telehealth was underutilized and understudied prior to the pandemic, and it was only with reduced regulations and increased payment parity that telehealth consultations saw a significant rise [43]. This underutilization and the limited studies on telehealth in specialty care such as SRH may explain why some HCPs in this study expressed hesitation and perceived a lack of knowledge, which contributed to their mistrust of telehealth’s effectiveness and adherence to ethical standards. Many providers advocated for a combination of virtual and in-person care. Additionally, the findings point to the need for patient orientation and education on how to seek sexual health services, as telehealth continues to be integrated into the healthcare system.

In contrast, agencies without existing telehealth infrastructure faced significant challenges in initiating telehealth for sexual and reproductive health (SRH) services. As a result, addressing typical SRH needs was delayed or halted altogether, as resources were redirected to focus on COVID-19 prevention and care activities. Over time, providers within these agencies began strategizing ways to meet patients’ health needs through telehealth and gradually started implementing these services. The attitude and knowledge of HCPs toward telehealth, coupled with strong collaborative relationships between physicians, advanced practice nurses, nurses, and social workers, played a crucial role in facilitating patients’ access to SRH care. These factors were essential in ensuring the continuity of care during a time of disruption [44].

Similar to findings in previous studies [19], the HCPs in this study reported the challenges with health insurance coverage for telehealth services particularly for modalities such as phone consultations. In such cases, the HCPs’ understanding and willingness to address patients’ healthcare needs became crucial. This commitment was commendable and aligned with the patient-centered sexual healthcare delivery framework advocated by Ohta, Ikeda and Sawa [45], Cuomo, Zucker and Dreslin [46], Gunaratne and Hansen [47] during the pandemic.

The successful implementation of telehealth was made possible by providers’ willingness to offer sexual health services, even when the facility faced financial losses due to reimbursement restrictions. These challenges, as reflected in the HCP narratives in this study and other reports [48], highlight the resilience of healthcare professionals in adapting to new service delivery models. Additionally, both patients’ and HCPs’ understanding of telehealth and its necessity helped make it a viable option, demonstrating the sustainability of this modality. Notably, 48 states including Washington, D.C., covered some form of telemedicine in 2016 compared to only 24 states in 2005, according to the American Telemedicine Association Coverage and Reimbursement report [49]. While telehealth reimbursement became a higher priority for widespread implementation during the pandemic, it was not a new concept, as policies and logistical frameworks had already been established nationally. Despite this increased national dialog on telehealth, both HCPs and patients were not well fully oriented to telehealth, and many needed more time to adjust due to the rapid adoption prompted by COVID-19 pandemic restrictions [48]. Therefore, there is a need for further research, along with additional provider and patient training and orientation, to build trust in telehealth and improve its application in SRH promotion and care [50].

An interdisciplinary approach and strong working relationship among the nurses, physicians and the social workers were also critical in facilitating implementation of telehealth services. Nurses supported various patient visits that did not require a physician’s attention, such as PrEP management and STI care, while social workers played a key role in following up with patients who missed appointments, ensuring STI treatment adherence, and facilitating partner treatment. This finding is consistent with previous studies, which noted that telehealth physicians often rely more on their support systems, particularly nurses, compared to physicians working in-person [43]. During the pandemic, nurses have been reported as crucial in ensuring the telehealth visits are well coordinated and that the workflow remains efficient [43]. Thus, nurses are essential in the delivery of telehealth-based care.

Additionally, the narratives from this study highlighted the critical role of social workers in the implementation of telehealth for SRH care. Previous research has shown that social workers bring essential skills and competencies, such as risk assessment, crisis management, advanced care planning, case management, systems navigation, problem-solving, resource allocation, and community mobilization, all of which were particularly important during the pandemic [51,52]. These findings emphasize the importance of a meaningful and mutually respectful interdisciplinary collaboration in achieving quality patient outcomes and ensuring the long-term success of telehealth services.

As in-person health services resume, health agencies should consider strategies to maintain telehealth services within their facilities. Providers in this study saw the merit for telehealth beyond COVID-19 pandemic restrictions. However, studies present differing opinions on whether telehealth is sustainable for delivering SRH services, with some suggesting that a hybrid model of remote and in-person care might be more feasible. For example, a previous study by Yelverton et al [19], questioned whether it was feasible for HIV testing services and PrEP to be provided entirely through telehealth and suggested a hybrid combination of remote and in-person delivery. The study also showed that participants questioned whether HIV home testing was done in a proper manner and stressed the need for in- person coaching of clients to ensure high-quality HIV testing and reliable results.

Hollander and Carr [10] highlight the importance of prioritizing telehealth when planning healthcare visits. They suggest that health facilities with existing telehealth services need to leverage them, while those lacking such services can outsource similar offerings to multinational telemedicine and virtual healthcare companies. Systems lacking such programs can outsource similar services to physicians and support staff provided by multinational telemedicine and virtual healthcare companies. Hollander and Carr [10] and Yelverton et al [19] recognize that a major barrier to large-scale telehealth adoption the challenges associated with HIV and STI screening This barrier could be overcome with better coordination of testing services and expanded testing sites. It is crucial to educate future HCPs about these social determinants so that they can work to mitigate the resulting disparities and thereby improve the overall health of patients and their communities.

### Implications

This study has implications for both practice and research. The study underscores the importance of recognizing SRH promotion as an essential service, particularly during pandemics. Healthcare systems must adopt measures that ensure the sustainability of telehealth for SRH promotion and care beyond the COVID-19 pandemic. These measures include expanding telehealth use for SRH, improving patient familiarity with technology, strengthening mail-order pharmacies, and distributing HIV and STI testing kits, interventions that have proven to be effective in Western-Central New York. Integrating these practices into routine care will enhance accessibility and continuity of SRH services via telehealth. HCPs should also adopt a patient-centered, multifaceted approach to SRH care, as demonstrated in New York State. This model, which emphasizes individualized, holistic care, has been shown to improve patient outcomes [45–47]. This study also highlights the need for further research to explore the integration of couple-based approach to care and telehealth into SRH services. Future studies should examine the effectiveness of these approaches in providing comprehensive care and reducing the transmission of HIV and other STIs. Research should also focus on strategies to improve the accessibility, fidelity, and equity of telehealth for SRH services, particularly in underserved areas, and evaluate long-term outcomes of telehealth for SRH and remote SRH interventions.

### Limitations

The HCPs in this study, including medical doctors and physician assistants, used the term "telemedicine" when reflecting on changes in healthcare modalities, while others primarily referred to it as "telehealth." While our findings focus on telehealth, it may be beneficial to expand the terminology to include e-health, m-health, and telemedicine. Doing so could broaden the scope of factors influencing utility and training needs for effective implementation, both in the U.S. and globally [14,42]. Furthermore, this study reports on the use of telehealth for sexual and reproductive health (SRH) promotion and care during the COVID-19 pandemic from the perspective of HCPs alone. Future research should explore telehealth for SRH promotion and care from the viewpoints of patients and service administrators to gain a more comprehensive understanding of its impact and effectiveness.

## Conclusion

This study highlights telehealth as a crucial tool for maintaining continuity of sexual and reproductive health (SRH) care, particularly for individuals facing barriers to access, especially when SRH services are not prioritized. Previous research indicates that even a slight reduction in HIV and STI screening can increase HIV infection rates by nearly 8% [53]. Therefore, the increased use in telehealth for SRH promotion and care must be prioritized in research and intervention development. These efforts are vital to addressing the SRH needs of all individuals, advancing the goal of ending the HIV epidemic, and reducing STI rates in Western-Central NY and across the US.
